# Choice of activation protocol impacts the yield and quality of CAR T cell product, particularly with older individuals

**DOI:** 10.1002/cti2.70016

**Published:** 2024-11-29

**Authors:** Palak H Mehta, Gemma S Trollope, Patrick Leung, Shivali Savita Chinni, Anna Iasinskaia, Aaron J Harrison, Hannah Hughes‐Parry, Misty R Jenkins, Michael H Kershaw, Anthony Jaworowski, Clare Y Slaney, Rachel M Koldej, David S Ritchie, Kylie M Quinn

**Affiliations:** ^1^ School of Health and Biomedical Sciences Royal Melbourne Institute of Technology (RMIT) Bundoora VIC Australia; ^2^ Peter MacCallum Cancer Centre Melbourne VIC Australia; ^3^ Sir Peter MacCallum Department of Oncology University of Melbourne Melbourne VIC Australia; ^4^ Immunology Division The Walter and Eliza Hall Institute of Medical Research Parkville VIC Australia; ^5^ Department of Medical Biology University of Melbourne Melbourne VIC Australia; ^6^ Department of Biochemistry and Chemistry, Institute of Molecular Science La Trobe University Bundoora VIC Australia; ^7^ Life Sciences Discipline Burnet Institute Melbourne VIC Australia; ^8^ Department of Infectious Diseases The Alfred Hospital and Monash University Melbourne VIC Australia; ^9^ Australian Cancer Research Foundation (ACRF) Translational Laboratory Royal Melbourne Hospital Melbourne VIC Australia; ^10^ Department of Medicine University of Melbourne Melbourne VIC Australia; ^11^ Department of Biochemistry, Biomedicine Discovery Institute Monash University Clayton VIC Australia

**Keywords:** ageing, CAR T cell, CD28, T cell activation

## Abstract

**Objectives:**

In clinical chimeric antigen receptor (CAR) T cell therapy, one of the strongest correlates of favorable patient responses is lower levels of differentiation in T cells from the peripheral blood mononuclear cell (PBMC) starting material or the CAR T cell product. T cells from older patients are inherently more differentiated, but we hypothesised that specific activation protocols could be used to limit CAR T cell differentiation during manufacturing, particularly in older patients.

**Methods:**

We used PBMCs from young (20–30 years old) and older (60+ years old) healthy donors to generate CAR T cells using two activation protocols: soluble anti‐(α) CD3 monoclonal antibody (mAb) *vs* immune complexes of αCD3 and αCD28 mAbs. Products were assessed for yield, function and differentiation, which was used as a measure of CAR T cell quality. T cells in PBMCs were assessed for CD28 expression and correlative analyses were performed.

**Results:**

Older samples generated fewer, more differentiated CAR T cells than young samples, and the αCD3/CD28 mAb protocol exacerbated this, further reducing yield and quality. CD28 expression by T cells correlated with CAR T cell differentiation, but T cell differentiation in PBMC starting material was a stronger correlate of CAR T cell differentiation.

**Conclusions:**

Choice of activation protocol can substantially impact on the yield and quality of CAR T cells during manufacturing. This is a key consideration for older patients whose samples already generate a poorer yield and lower quality of CAR T cells.

## Graphical abstract

It is unclear whether specific T cell activation protocols are more or less effective for CAR T cell therapy in older patients. Our study demonstrates that specific activation protocols (anti‐CD3/28 mAb complexes) can exacerbate low yield and advanced differentiation already seen in older patients and generate a poorer quality CAR T cell product.

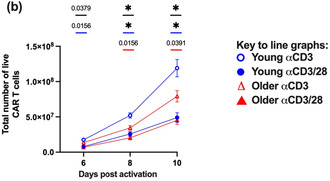

## Introduction

Chimeric antigen receptor T cell therapy (CTT) is a potent treatment for haematological cancers.^1^, ^2^, ^3^, ^4^ Currently, commercially manufactured CAR T cells are generated by isolating peripheral blood mononuclear cells (PBMCs) from patients and activating their T cells *in vitro* to support retroviral or lentiviral transduction with the CAR transgene. These CAR T cells are further expanded *in vitro* before reinfusion into the patient where, ideally, they undergo additional expansion, eliminate malignant cells, and establish long‐lived memory populations that can maintain anti‐tumor surveillance.

T cell activation is therefore a critical step in CAR T cell manufacturing protocols. Full activation of naïve T cells *in vivo* requires three signals: antigenic signalling via the T cell receptor (TCR), signalling through a costimulatory receptor such as CD28, and cytokine stimulation primarily driven by interleukin (IL)‐2.^5^ Commercial CAR T cell manufacturing protocols typically trigger T cell activation with either soluble αCD3 mAb^4^ or bead‐immobilised or complexed αCD3 and αCD28 mAbs,^3^ both in the presence of recombinant IL‐2, although interest is growing in alternative activation protocols. Optimal T cell activation for CTT should drive proliferation to support transduction and generation of sufficient CAR T cells.^5^, ^6^ However, optimal activation should also minimise activation‐induced cell death (AICD) and minimise excessive T cell differentiation in the CAR T cell product.

Minimising T cell differentiation is important as, currently, the degree of differentiation observed either in T cells in the PBMC starting material or in the CAR T cell product is the strongest predictor of CTT efficacy. A study by Garfall and colleagues demonstrated that a higher frequency of less differentiated cells (CD8^+^CD45RO^−^CD27^+^) in isolated PBMCs correlated with more proliferation and better response rates for CTT.^7^ A study by Fraietta and colleagues also showed that a higher frequency of less differentiated cells, such as naïve T (T_N_) cells, stem cell memory T (T_SCM_) cells or central memory T (T_CM_) cells in the CAR T cell product correlated with successful CTT.^8^ A study by Fischer and colleagues similarly showed that a higher frequency of T_CM_ cells correlated with more CAR T cell expansion and better disease control in multiple myeloma patients.^9^ Higher frequencies of T_N_,^10^ T_CM_ ^11^ and/or T_SCM_ ^12^, ^13^ cells support improved expansion, engraftment and/or tumor control in human and animal studies, likely because these cells are more proliferative and functionally flexible than more differentiated subsets. In T cell biology, the degree of differentiation is referred to as the ‘quality’ of these cells. High‐quality T cells exhibit less differentiation, make a wider combination of cytokines, are longer‐lived *in vivo* and respond more robustly to restimulation,^14^ all of which is thought to support better CAR T cell efficacy.^9^ Accordingly, in this study, we refer the degree of differentiation of T cells or CAR T cells as their quality. However, this is distinct from quality control assessments commonly used during commercial CAR T cell manufacturing, such as sterility, identity, specificity and potency assays.^15^

The age of the patient directly impacts the degree of differentiation in T cells in the PBMC starting material and could therefore also impact the degree of differentiation in the CAR T cell product. Ageing leads to an accumulation of more differentiated antigen‐experienced T cell subsets, including subsets with reduced proliferative capacity, such as effector memory T (T_EM_) cells or effector memory that re‐express CD45RA T (T_EMRA_) cells.^16^, ^17^, ^18^, ^19^ Critically, ageing also leads to decreased expression of CD28 on T cells, particularly within the CD8 compartment, with suggestions that this is because of repeated antigen stimulation or chronic type I interferon (IFN) exposure.^20^, ^21^ As a result, we predict that older patients would (i) have more differentiated T cells in their PBMC starting material, (ii) respond poorly to CD28 stimulation, (iii) have more differentiated CAR T cell product and (iv) potentially have a reduced rate of response to CTT.

To define how distinct activation protocols impact on CAR T cell generation with older patients, we generated CAR T cells using PBMC samples from young or older adult donors. To activate donor T cells, we used either soluble αCD3 mAb or complexes of αCD3 and αCD28 mAbs and compared early T cell activation/proliferation and CAR T cell yield, quality, and function (cytokine production, killing capacity). We also defined a new metric, the differentiation score, to measure net degree of differentiation within a T cell population more comprehensively and we used this as a proxy for CAR T cell product quality. Our data demonstrates clear age‐ and activation protocol‐specific effects that culminate in older, αCD3/28 mAb activated samples yielding fewer, poorer quality CAR T cells than those in younger samples and αCD3 mAb activated samples. These cells also trend towards expressing more IFNγ and tumor necrosis factor (TNF). CD28 expression was lower in some older individuals and correlated modestly with the differentiation score of the CAR T cell product, but the differentiation score of T cells in the PBMC starting material was a stronger correlate. Collectively, this highlights that T cell quality in PBMC starting material has a dominant effect on the quality of the CAR T cell product, but specific activation protocols could be used to modulate CAR T cell quality, which may be particularly important for older patients.

## Results

### Ageing leads to more differentiated T cells in PBMC starting material

**Figure 1:**
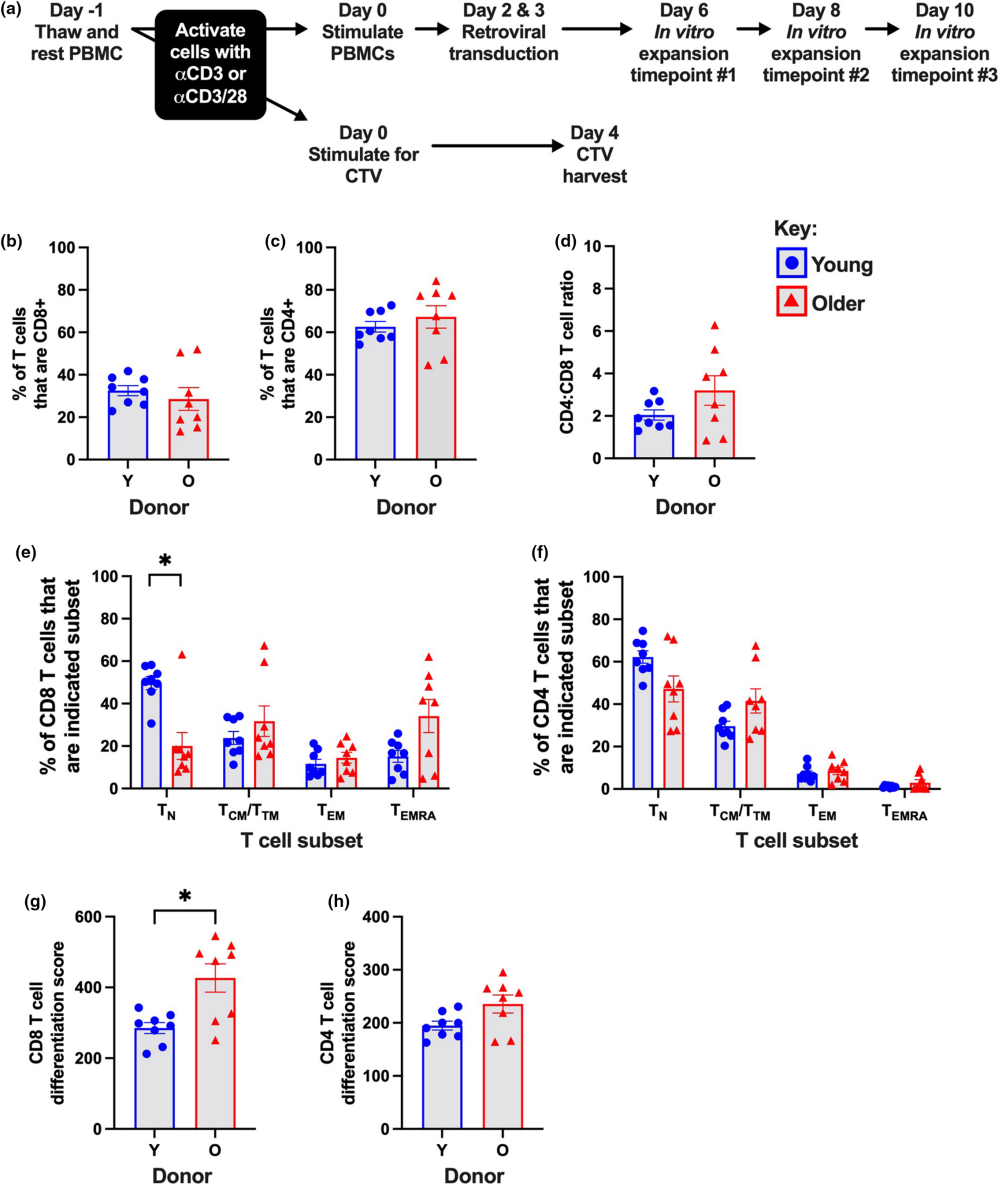
T cell characteristics in PBMCs isolated from healthy young and older donors. **(a)** Schematic diagram of key experimental steps and time points of analysis. Frequency of CD3^+^ T cells that are either **(b)** CD8^+^, or **(c)** CD4^+^, and the resulting **(d)** CD4:CD8 T cell ratio. Frequency of **(e)** CD8 or **(f)** CD4 T cells that are the indicated subset. The differentiation score of **(g)** CD8 or **(h)** CD4 T cells in PBMC starting material. Bars indicate mean ± the standard error of the mean (SEM), and symbols indicate donors. *P ≤ 0.05, using the Mann–Whitney *U*‐test.

To define key characteristics of the starting material for CAR T cell generation, we isolated PBMCs from healthy young (range 23–30 years; median age 26 years) and older (range 62–69 years; median age 64 years) adult donors prior to commencing the CAR T cell protocol (Figure 1a). We performed flow cytometry and assessed the relative abundance and differentiation phenotype of CD4 and CD8 T cells. Many, but not all, older individuals had lower frequencies of CD8 T cells, the frequency of CD4 T cells were unaltered (Figure 1b and c) and the CD4:CD8 ratio trended up with age (Figure 1d), which is often observed in older individuals.^22^ When shifts in CD4 and CD8 T cell differentiation were assessed across T_N_, T_CM_/transitional memory (T_TM_), T_EM_ and T_EMRA_ subsets, the frequency of CD8 T cells that were T_N_ phenotype was significantly reduced in older donors, with a trend towards an increase in T_EMRA_ phenotype cells and similar trends in CD4 T cells (Figure 1e and f), consistent with previous observations in older individuals.^16^, ^17^ Our donor cohort, therefore, exhibits age‐related differences that are commonly seen in older individuals, namely a reduction in circulating CD8 T cells, loss of naïve T cells and accumulation of antigen‐experienced T cells.

Lower frequencies of differentiated T cells in PBMC starting material are one of the strongest correlates of subsequent CAR T cell efficacy.^7^, ^8^, ^9^ However, measuring the frequency of one subset does not account for the full spectrum of differentiation encompassing T_N_, T_CM_/T_TM_, T_EM_ and T_EMRA_ cells. We therefore created a novel metric called the ‘differentiation score’ which measures cumulative differentiation progression in a population of T cells. The most differentiated T cell subset, T_EMRA_ cells, was weighted the highest, followed by T_EM_, T_CM_/T_TM_ and lastly T_N_ cells, weightings were multiplied by the frequency of each subset and scores were summed to provide a single metric. The differentiation score was significantly higher for CD8 T cells and trended higher for CD4 T cells in PBMCs from older as compared to younger donors (Figure 1g and h). This reinforces that T cells from older patients are more differentiated and suggests that the differentiation score can be used as an integrated measure of T cell differentiation.

### Ageing and activation protocol choice can reduce CAR T cell yield

**Figure 2:**
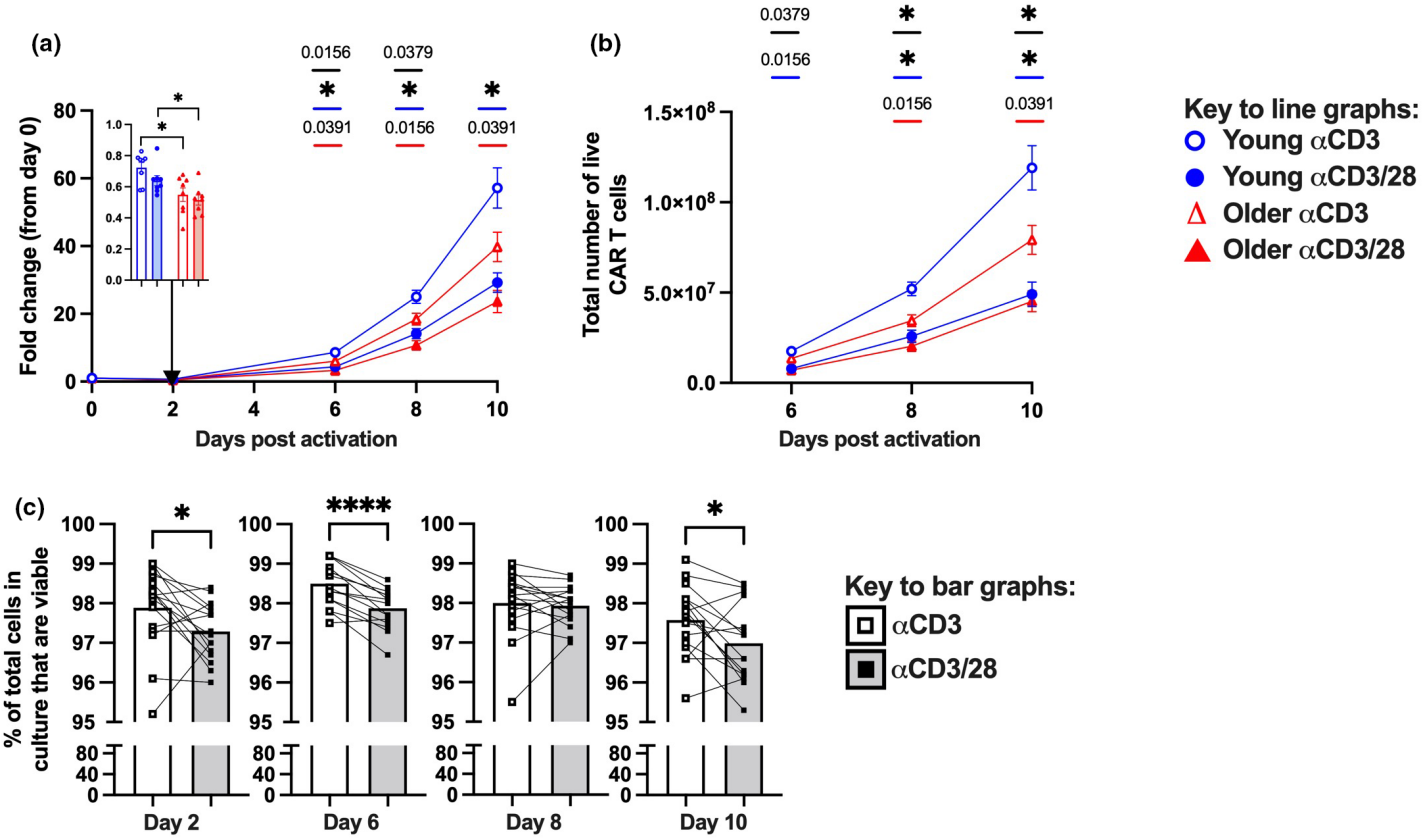
Impact of activation protocol and age on *in vitro* generation of CAR T cells. **(a)** Fold change in the number of total live leukocytes relative to day 0, with fold change at day 2 in the inset bar graph. **(b)** Total number of live CAR T cells recovered at the indicated day after activation. **(c)** Frequency of total cells that are viable at the indicated day after activation. Bars or symbols on the line graphs indicate mean ± SEM, and symbols on bar graphs indicate donors. *P ≤ 0.05 **(c)** or *P* ≤ 0.0125 **(a, b)** and **** *P* ≤ 0.0001 **(c)** using the Mann–Whitney *U*‐test (between donor groups) or pairwise Wilcoxon test (between activation protocols) with Bonferroni correction for multiple comparisons. **(a, b)** *P* ≤ 0.05 is indicated on the graphs in **(a)** and **(b)**, where black bars indicate young *vs* older with the αCD3 mAb protocol, red bars indicate comparison of young with αCD3 *vs* αCD3mAb protocols, and blue bars indicate comparison of older with αCD3 *vs* αCD3mAb protocols.

To define how patient age and activation protocol impacts on CAR T cell generation, we performed a standard CAR T cell manufacturing protocol on PBMC samples from young and older donors, and measured cell yield, viability and CAR expression after activation (Figure 1a). We compared two activation protocols: soluble αCD3 mAb and complexed αCD3/28 mAbs (ImmunoCult™ reagent). Samples from older donors generated fewer total live cells and yielded lower numbers of CAR T cells during the protocol than samples from young donors (Figure 2a and b). The age‐related difference in cell number was apparent by day 2 after activation (Figure 2a insert). The αCD3/28 mAb protocol further exacerbated this. In young donors, the αCD3/28 mAb protocol consistently generated fewer total live cells and fewer CAR T cells than the αCD3 mAb protocol, with similar trends seen in samples from older donors (Figure 2a and b).

Different rates of T cell death and/or proliferation could contribute to differences in CAR T cell numbers. The αCD3/28 mAb protocol modestly reduced cell viability compared to the αCD3 mAb protocol across most time points (Figure 2c). It also modestly impacted proliferation as measured by a parallel CTV assay, with a trend towards an increase in CD8 and a significant decrease in CD4 T cell mean number of divisions at day 4 after activation compared to the αCD3 mAb protocol (Supplementary figure 1a, b). Changes in T cell activation can also impact CAR transduction rates.^23^ When CAR expression was assessed at day 10, the αCD3/28 mAb protocol modestly decreased the frequency of CD8 or CD4 T cells that were CAR+ as compared to the αCD3 mAb protocol (Supplementary figure 1c, d). It should be noted however that CAR expression at day 10 is a function of CAR transduction, CAR T cell proliferation and survival. Collectively, this suggests that the αCD3/28 mAb protocol promotes cell death, with more modest and/or mixed effects on proliferation.

### Ageing and activation protocol choice can increase CAR T cell product differentiation

**Figure 3:**
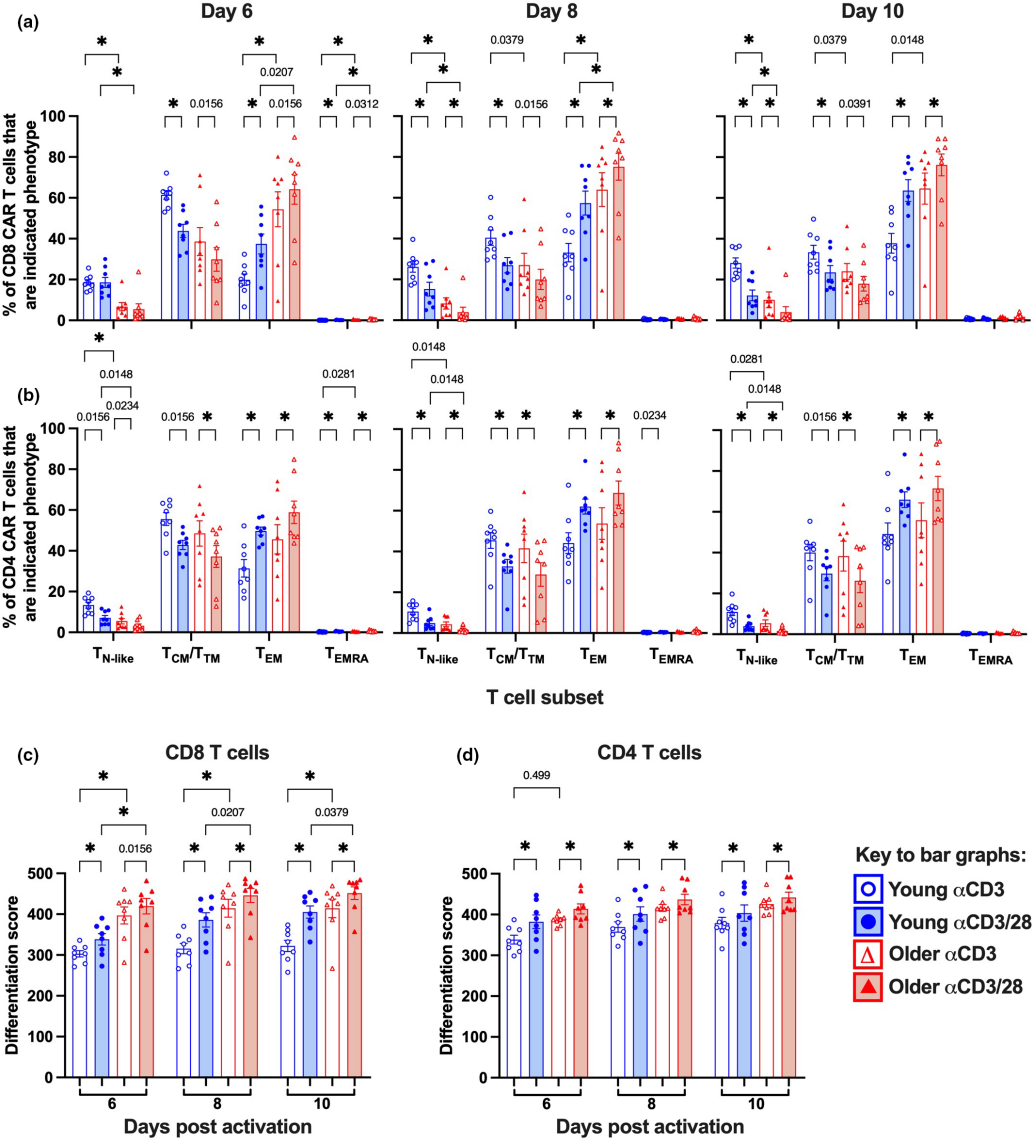
Impact of activation protocol and age on the differentiation and quality of CAR T cells. Frequency of **(a)** CD8 or **(b)** CD4 CAR T cells that are the indicated subset at days 6, 8 and 10 after activation. Differentiation score of **(c)** CD8 and **(d)** CD4 T cells for CAR T cells at days 6, 8 and 10 after activation. Bar graphs indicate mean ± SEM, symbols indicate donors, *P ≤ 0.0125 using the Mann–Whitney *U*‐test (between donor groups) or pairwise Wilcoxon test (between activation protocols) with Bonferroni correction for multiple comparisons. If *P* ≤ 0.05, we have indicated this on the graphs.

Given that CD28 co‐stimulatory signals are needed for optimal activation of naïve T cells,^24^ we hypothesised that the αCD3/28 mAb protocol would enhance the recruitment of naïve T cells and generate a more differentiated CAR T cell product. Samples from older donors generated a markedly more differentiated CAR T cell product than young samples (Figure 3a and b), consistent with their PBMC starting material (Figure 1e and f). Additionally, the αCD3/28 mAb protocol was seen to generate more differentiated CAR T cells than the αCD3 mAb protocol in both young and older samples and at all time points analysed during expansion (Figure 3a and b). Specifically, the αCD3/28 mAb protocol generated fewer T_N_‐like cells and T_CM_/T_TM_ cells, and more T_EM_ cells, particularly in CD8 CAR T cells (Figure 3a and b). In contrast, CD8 and CD4 T_N_‐like cells were better retained and even increased in proportion during expansion with the αCD3 mAb protocol in both donor groups (Supplementary figure 2a, b). Differentiation scores were also significantly higher in older donors than those in young donors for CD8 and CD4 CAR T cells across all time points (Figure 3c and d), and the αCD3/28 mAb protocol further increased differentiation scores for young and older donors in CD8 CAR T cells (Figure 3c and d). Collectively, this demonstrates that the αCD3/28 mAb protocol significantly decreases the overall quality of the CAR T cell product, which could compromise CAR T cell product efficacy especially for older donors.

### Ageing and activation protocol choice can increase CAR T cell functional markers

To define how age and activation protocols impact CAR T cell function, we measured cytokine production and cytotoxic capacity of the CAR T cell products.

**Figure 4:**
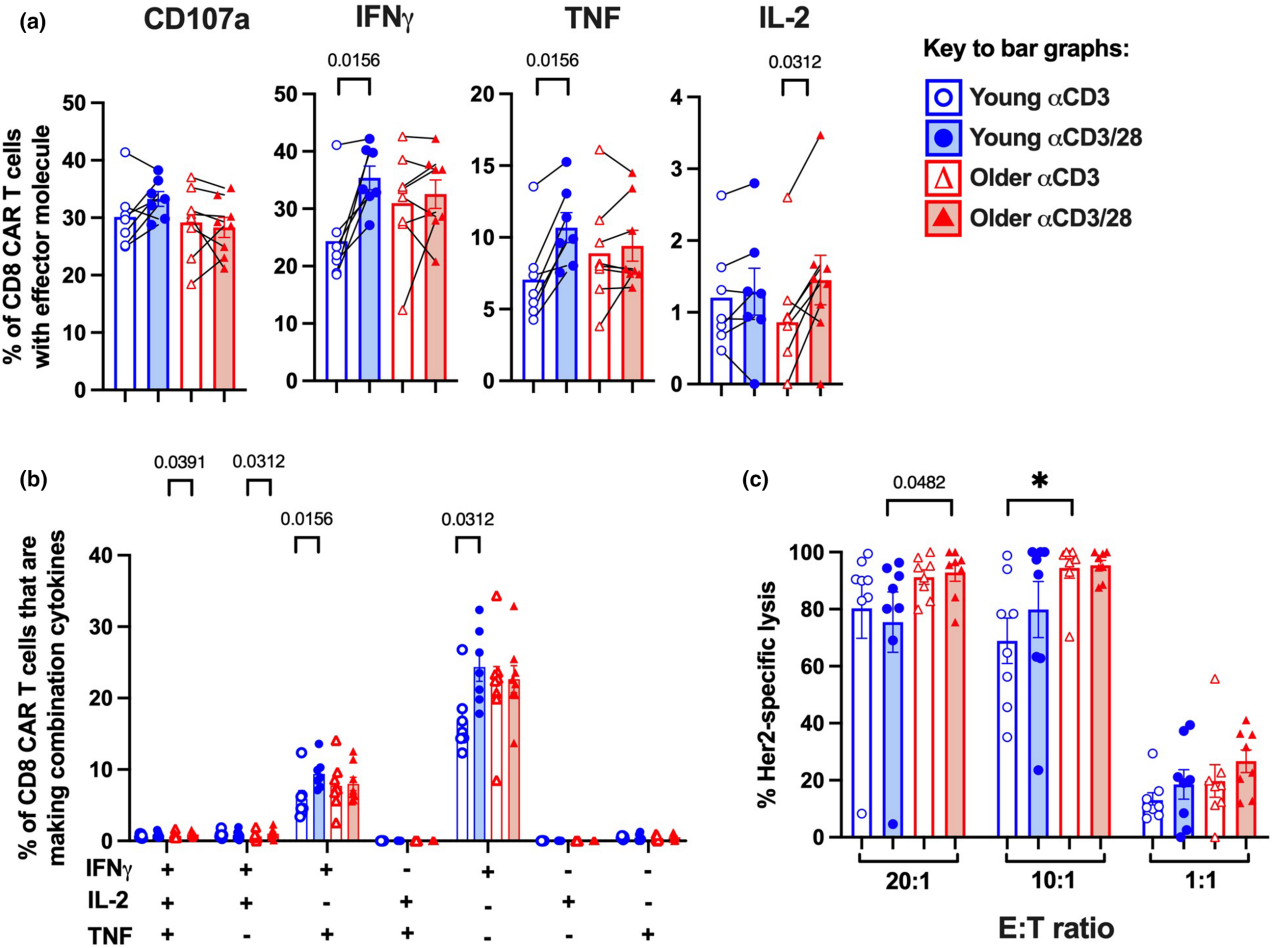
Impact of activation protocol and age on the functionality of the CAR T cells. **(a)** Frequency of CD8 CAR T cells that express CD107a, IFNγ, TNF or IL‐2 in response to HER2. **(b)** Frequency of CD8 CAR T cells that express any combination of IFNγ, TNF and/or IL‐2 in response to HER2. **(c)** Percent HER2‐specific lysis of target cells at E:T ratios of 20:1, 10:1 and 1:1. Bars indicate mean ± SEM, symbols indicate donors, *P ≤ 0.0125 using the Mann–Whitney *U*‐test (between donor groups) or pairwise Wilcoxon test (between activation protocols) with Bonferroni correction for multiple comparisons. If *P* ≤ 0.05, we have indicated this on the graphs.

To assess cytokine production, we co‐cultured CAR T cells overnight with iK562 cells that express HER2, and then performed intracellular cytokine staining to measure the cytotoxic cytokines, IFNγ and TNF, and the pro‐proliferation cytokine, IL‐2. An antibody for CD107a was included in the co‐cultures to detect degranulation. There were strong trends towards increased frequencies of CD8 CAR T cells expressing IFNγ or TNF in young donors only (Figure 4a). Many older donors already exhibited elevated frequencies of CD8 CAR T cells expressing IFNγ, and the αCD3/28 mAb protocol did not increase this further (Figure 4a). The αCD3/28 mAb protocol trended towards higher frequencies of CD8 CAR T cells that expressed IL‐2 in older donors only (Figure 4a). When the combination of cytokines being expressed was assessed as an alternative measure of T cell quality,^14^ the αCD3/28 mAb protocol again trended towards more terminally differentiated subsets that express IFNγ and TNF, or IFNγ alone in young donors (Figure 4b). CD4 T cells did not exhibit marked age‐ or activation protocol‐related differences in cytokine production (Supplementary figure 3). The αCD3/28 mAb protocol therefore may augment expression of cytotoxic effector molecules and terminal differentiation, particularly in young donors.

To directly measure cytotoxic capacity, we cocultured CAR T cells overnight with a target tumor cell line, MDA‐468 expressing HER2 and luciferase, at 20:1, 10:1 or 1:1 effector‐to‐target ratios and assessed percent‐specific lysis via a luminescence assay. CAR T cells generated from older donors were significantly more cytolytic than young donors with the αCD3 mAb protocol at the 10:1 ratio and similarly trended towards being more cytolytic with the αCD3/28 mAb protocol at the 20:1 ratio (Figure 4c), which was consistent with the cytokine data. There were no statistically significant differences between the αCD3 mAb and αCD3/28 mAb protocols in terms of CAR T cells cytotoxicity (Figure 4c). This suggest that increased age is a major driver of increased cytotoxicity in CAR T cell product.

### T cell CD28 expression or differentiation is predictive of CAR T cell product differentiation

Ageing has previously been reported to lead to reduced CD28 expression in T cells, with a more pronounced effect on CD8 than that in CD4 T cells,^20^ and some suggestions that CD28 is downregulated to limit non‐specific responses to co‐stimulatory signals.^25^ We aimed to validate these age‐related shifts in CD28 expression in our cohort.

**Figure 5:**
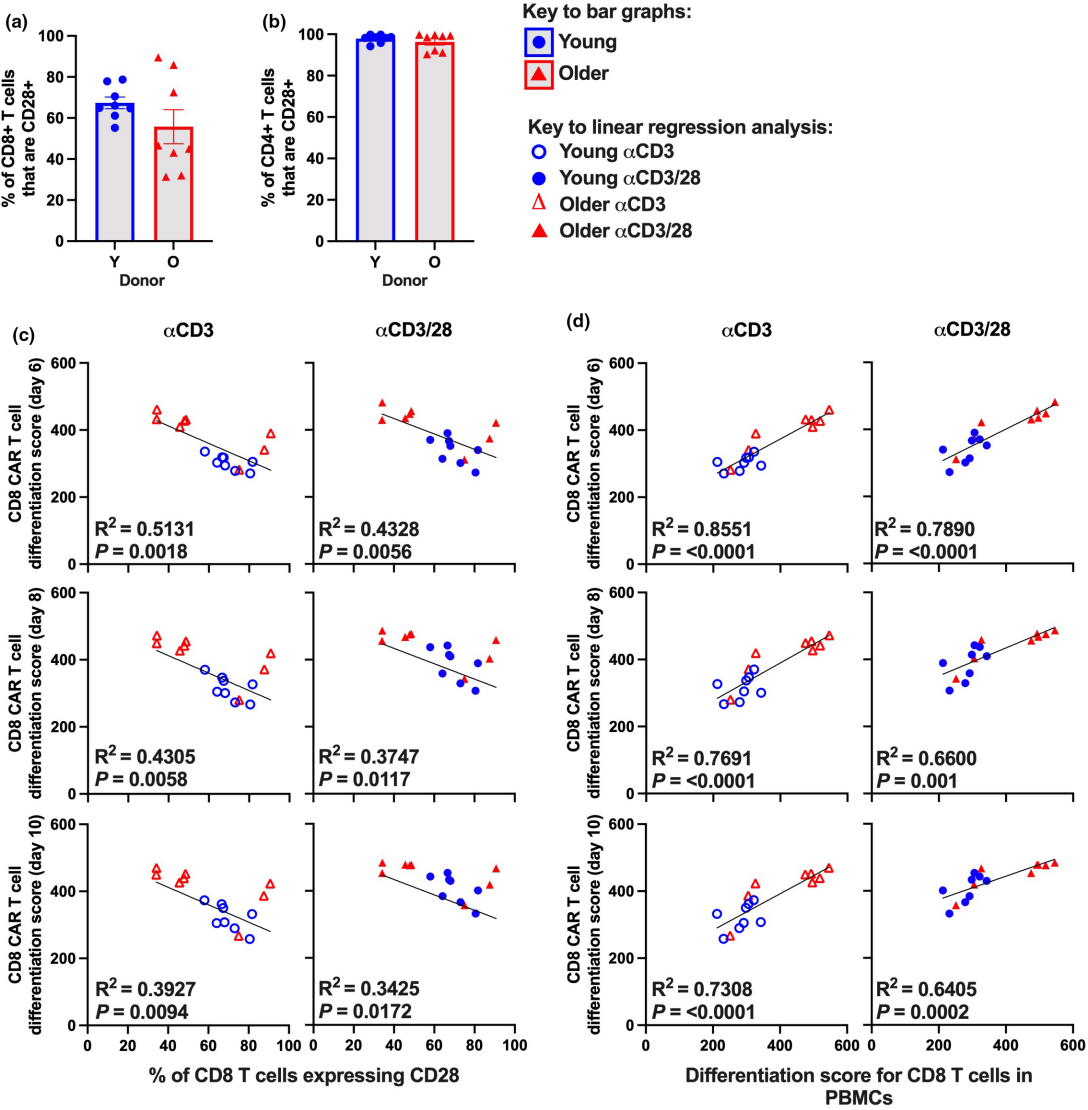
CD28 expression or differentiation score of T cells in PBMCs as predictive biomarkers for CAR T cell quality. Frequency of **(a)** CD8 or **(b)** CD4 T cells that express CD28. Simple linear regression plots showing **(c)** the frequency of CD8 T cells expressing CD28 or **(d)** the differentiation score of CD8 T cells in PBMC starting material *vs* the differentiation score of CD8 CAR T cells at days 6, 8 and 10 after activation. Bars indicate mean ± SEM, symbols indicate donors using the Mann–Whitney *U*‐test. *R* ^2^ values are indicated within each linear regression panel and *P*‐values were calculated by linear regression analysis.

We first assessed CD28 expression on T cells in PBMC starting material by flow cytometry. Most but not all older individuals had lower frequencies of both CD8 and CD4 T cells that express CD28 (Figure 5a and b). Further analysis revealed that age did not alter the frequency of cells in each subset that express CD28 or the MFI of CD28 on CD28^+^ cells in each subset (Supplementary figure 4). Notably, regardless of donor age, the frequency of cells expressing CD28 was highest in T_N_ and T_CM_/T_TM_ subsets, but the CD28 expression level was highest on T_CM_/T_TM_ subset (Supplementary figure 4). This demonstrates that age‐related reductions in CD28 expression on bulk CD8 and CD4 T cells are driven by changes in T cell subset composition (fewer CD28^int^ T_N_ cells, more CD28^−^ T_EM_ and T_EMRA_ cells), rather than changes in CD28 expression on individual cell subsets.

We then tested whether the frequency of T cells expressing CD28 in PBMC starting material correlated with the differentiation score of the resulting CAR T cell product using simple linear regression analysis. These measures did correlate significantly but not strongly for CD8 T cells for both activation protocols at 6 days after activation (αCD3 mAb *P* = 0.0018, *R* ^2^ = 0.5131; αCD3/28 mAb *P* = 0.0056, *R* ^2^ = 0.4328) (Figure 5c). We then assessed whether the differentiation score of T cells in the PBMC starting material correlated with the differentiation score of the resulting CAR T cell product. These measures correlated significantly and strongly, particularly for CD8 T cells with the αCD3 mAb protocol at 6 days after activation (αCD3 mAb *P* = < 0.0001, *R* ^2^ = 0.8551; αCD3/28 mAb *P* = < 0.0001, *R* ^2^ = 0.7890) (Figure 5d). However, the predictive strength of CD28 expression or differentiation score of T cells in PBMC starting material for the differentiation score of CD8 CAR T cells decreased with increasing time in culture (Figure 5c and d). This highlights that the quality of T cells in the PBMC starting material is highly predictive for the quality of the CAR T cell product, but predictive power may decrease with time in culture.

## Discussion

Our study aimed to define how different activation protocols impact the generation of CAR T cells specifically with samples from older individuals. We showed that older individuals, in general, generated fewer CAR T cells and a more differentiated CAR T cell product. Critically, the αCD3/28 mAb protocol further reduced the overall CAR T cell yield, decreased viability, and exacerbated differentiation in the CAR T cell product. Our work and others have shown that older patients have more differentiated PBMC starting material^16^, ^17^, ^18^, ^19^ with reduced frequencies of CD28 expressing T cells.^20^ More differentiated CAR T cell products correlate with poorer efficacy of CTT in patients.^7^, ^8^, ^9^ Our study therefore suggests that an αCD3/CD28 mAb protocol yields fewer, poorer quality, potentially less efficacious CAR T cells than those in an αCD3 mAb protocol, especially for older patients.

To understand how the αCD3 mAb and αCD3/CD28 mAb protocols generate distinct CAR T cell products, it is important to note that there are three key variables between these two conditions. Firstly, the αCD3 mAb protocol uses a soluble form of the mAb, while the αCD3/CD28 mAb protocol uses a complexed form, which increases binding avidity and is known to increase T cell activation.^26^ Secondly, the αCD3/CD28 mAb protocol includes a costimulatory signal in the form of the αCD28 mAb, which is also known to amplify TCR signalling strength.^27^ Thirdly, the absolute amount of αCD3 mAb may differ across our two activation protocols. We used a concentration of soluble αCD3 mAb that has been used in production of other clinical CAR T cell products by our broader team^28^ but we are unable to define the amount of αCD3 mAb in the αCD3/28 mAb immune complexes as this is a commercial product and the manufacturer cannot disclose this proprietary information. The impact of this final variable therefore remains unresolved.

However, our data are consistent with key outcomes from a study by Li and Kulander that directly compared the impact of soluble αCD3 mAb with bead‐bound αCD3/CD28 mAbs during *in vitro* T cell activation.^5^ In their study, activating with αCD3/CD28 mAbs increased the expansion rate of CD4 but not CD8 T cells and increased the differentiation of CD8 but not CD4 T cells.^5^ In contrast, our study focused on CAR T cell generation and showed that activating with αCD3/CD28 mAbs very modestly increased the division rate of CD8 but led to lower recovery of both CD4 and CD8 CAR T cells, along with increased differentiation of these cells. While it is not clear why these studies have different outcomes regarding cell expansion, both studies show that CD28 stimulation increases the differentiation of the resulting T cells or CAR T cells.

These datasets are also consistent with a recent clinical study by Bachy and colleagues.^29^ The first commercial clinical CAR T cell products, axicabtagene ciloleucel (Yescarta®; axi‐cel) and tisagenlecleucel (Kymriah®; tisa‐cel), differ in their transduction approaches (retroviral *vs* lentiviral) and the costimulatory domain within the CAR constructs (CD28 *vs* 41BB), but they also differ in their activation protocols. Axi‐cel uses soluble αCD3 mAb^4^ while tisa‐cel uses bead‐bound αCD3 and αCD28 mAbs,^3^ both in the presence of IL‐2. Bachy and colleagues compared the efficacy of axi‐cel *vs* tisa‐cel products in a recent retrospective clinical study with a large cohort of patients with relapsed/refractory diffuse large B cell lymphoma (DLBCL) and a median age of 63 years.^29^ Patients who received axi‐cel not only had considerably higher rates of severe cytokine release syndrome but also they had higher rates of complete response (CR; 60–80% with axi‐cel *vs* 44–60% with tisa‐cel) and higher rates of 1‐year progression‐free survival (PFS; 46.6% with axi‐cel *vs* 32.2% with tisa‐cel).^29^ It has been largely assumed that differences in the costimulatory domains are driving these differences in clinical efficacy.^29^, ^30^ However, our data suggest that the choice of activation protocol may also contribute, as an αCD3/CD28 mAb protocol significantly exacerbated CAR T cell differentiation in older patients as compared to an αCD3 mAb protocol, and increased CAR T cell differentiation has been seen to correlate with poorer CAR T cell efficacy.^7^, ^8^, ^9^

Our study also demonstrates that the degree of T cell differentiation seen in the PBMC starting material correlates with the degree of differentiation of the CAR T cell product. However, this correlation was strongest at day 6 after activation, which is relatively early in the *in vitro* expansion protocol, and the strength of correlation (*R* ^2^ values) declined with time after activation for both αCD3 and αCD3/28 mAb protocols. This decline in the strength of correlation with increasing time in culture also occurred when CD28 expression on CD8 T cells was used as a variable. This suggests that if we try to use these types of metrics as prognostic tools, they may be more predictive with CTT protocols that use shorter as opposed to longer *in vitro* expansion protocols. With the recent success of the YTB323 CAR T cell product that uses a 2‐day *in vitro* activation protocol, wider clinical use of shortened CTT protocols may yield both more efficacious and more predictable CAR T cell products.^31^

Of note, our study has several key limitations. Firstly, we assessed the impact of age on correlates of CTT efficacy rather than directly on CTT efficacy. We focused on the differentiation state of T cells as the current gold standard for predicting CTT efficacy^7^, ^8^, ^9^, ^13^ and developed a metric called the differentiation score to aggregate differentiation across all T cell subsets. However, this novel metric remains to be validated as a predictor of CTT efficacy specifically. Secondly, we only compared αCD3 mAb and αCD3/28 mAb activation protocols using a second‐generation CAR containing a CD28 signalling domain and retroviral transduction. It remains to be seen whether these age‐ and activation protocol‐related differences are still seen with other CAR constructs, other costimulatory signalling domains and lentiviral transduction. Finally, chronic infections such as cytomegalovirus or Epstein–Barr virus can promote T cell differentiation with marked clonal expansions.^32^ This study has not specifically assessed the impact of chronic infections as distinct from ageing, as the majority of older Australians will be sero‐positive for both of these pathogens.^33^ It is possible that chronic infections contribute to the observed age‐related changes in CAR T cell generation.

While this study compared αCD3 mAb and αCD3/28 mAb activation protocols, there are many alternative activation protocols that could be considered for older patients. Other forms of co‐stimulation in addition to or instead of CD28 could potentially improve CAR T cell product quality in older patient groups. For example, a study by Zhu and colleagues demonstrated that the addition of a αCD137 mAb significantly enhances the expansion of CD8 memory subsets in mouse models.^34^ The impact of other costimulatory signals such as ICOS or CD27^35^, ^36^ should also be assessed specifically in older patients. Alternative cytokines could be used, such as IL‐7, IL‐15 and IL‐21, in CAR T cell cultures to limit T cell differentiation.^37^, ^38^ Finally, titrating the amount of αCD3 mAb, αCD28 mAb and/or IL‐2 should be evaluated with older patient groups. Current T cell activation strategies tend to use relatively high doses of all these reagents which could lead to T cell hyperactivation, AICD and exacerbated differentiation,^5^ particularly in older patients with more differentiated PBMC starting material.

Ultimately this study shows that, while CTT enables us to use a patient's own T cells as a potent anti‐cancer treatment, the intrinsic fitness of these T cells can change substantially with age. T cells from older patients are more differentiated, express lower levels of CD28,^20^ and are intrinsically more difficult to activate^17^ but rational selection of activation protocols for older patients could mitigate these challenges. With greater insight into how factors like patient age impact CTT, we could profile the specific biology of an individual patient using metrics such as the differentiation score, and select an appropriate CAR T cell modality to match this biology.

## Methods

### PBMC samples, CAR T cell samples and cell lines

Peripheral blood mononuclear cells were isolated from buffy packs collected from healthy young (20–30 years of age; 26 years of median age, *n* = 8, four females, four males) and older donors (60–70 years of age; 62 years of median age, *n* = 8, five females, three males) provided by the Australian Red Cross Blood Service under ethics approval (Project # 81‐19/22437) from the RMIT College of Human Ethics Advisory Network.

PBMCs were isolated from buffy packs using a standard Ficoll gradient approach. PBMC and CAR T cell products were cryopreserved in freezing media (10% DMSO in foetal calf serum (FCS)) and stored at ≤ −150°C until use. Cryopreserved PBMCs or CAR T cells were thawed and rested in complete RPMI (RPMI supplemented with 10% FCS, 100 IU mL^−1^ penicillin/streptomycin, 1 mM sodium pyruvate, 2 mM glutamine and 100 μM MEM non‐essential amino acids) at 37°C in 5% CO_2_ for 16 h before experimental use.

K562 cells that express non‐signalling (ns) human epidermal growth factor receptor 2 (HER2) (nsHER2^+^) or did not express nsHER2 (nsHER2^−^) were used for antigen‐driven stimulation of HER2‐specific CAR T cells. K562 cells were irradiated (iK562) to an absorbed dose of ~50 Gy using megavoltage x‐rays from the Elekta Versa linear accelerator 6 MV photon beam at ARPANSA, then cryopreserved until use in CAR T cell stimulation.^39^

### Vector production and generation of CAR T cells

Human epidermal growth factor receptor 2‐specific CAR T cells were generated using a previously described method^28^ (Figure 1a). Briefly, a retroviral packaging cell line (PG13) was cultured for a minimum of 7 days to produce retroviral vector containing the transgene that encodes a HER2‐specific CAR (scFv clone FRP5)^40^ with a CD28/CD3ζ intracellular signalling domain.^41^ PBMCs were thawed and rested, then 5 × 10^6^ PBMCs were plated and T cells were activated with either soluble αCD3 mAb (OKT3, WEHI Antibody Production Facility) at a final concentration of 0.03 μg mL^−1^, or with αCD3 and αCD28 mAb antibody complexes (ImmunoCult™ Human CD3/CD28 T Cell Activator (StemCell Technologies, Vancouver, Canada)) used at final volume of 12.5 μL mL^−1^. cRPMI and recombinant human IL‐2 (rhIL‐2) (600 IU mL^−1^; Peprotech/ThermoFisher Scientific, Waltham, USA) were used in all cultures, with incubation at 37°C and 5% CO_2_. At days 2 and 3 after activation, cultures were transduced twice with retroviral vector containing HER2‐specific CAR, using RetroNectin (TakaraBio, Kyoto, Japan) and spinoculation. T cells were replated and expanded for up to 10 days after activation, with refreshment of rhIL‐2 every 2 days. Total live cell counts during culture were obtained with the Countess™ 2 cell counter (Life Technologies/ThermoFisher Scientific, Waltham, USA) and 0.4% trypan blue solution (Gibco/ThermoFisher Scientific, Waltham, USA).

### CD28 expression and T cell differentiation in PBMCs and CAR T cells

To define T cell differentiation in PBMC starting material or CAR T cell products, approximately 1 × 10^6^ thawed and rested cells were stained for viability and surface markers. Cells were stained first with LIVE/DEAD™ Near‐IR Dead Cell Stain (Life Technologies) for 10 min at room temperature, then stained with fluorescently conjugated anti‐human mAbs against the following surface markers for 30 min at 4°C: TCR (clone IP26; AF700; eBiosciences/ThermoFisher Scientific, Waltham, USA), CD8 (SKI; SB600; eBiosciences), CD4 (SK3; APC; Tonbo Biosciences/Cytek, Fremont, USA), CD45RA (5H9; FITC; BD Pharmingen, Franklin Lakes, USA), CD27 (L128; BV711; BD Pharmingen), CD28 (CD28.2; PE; BD Pharmingen). Additionally, HER2‐specific CAR was detected in CAR T cell products with a Flag‐Tag mAb (L5; PE‐Cy7; BioLegend, San Diego, USA). The gating strategy is detailed in Supplementary figure 5. T_N_ cells are defined to be CD45RA^hi^CD27^hi^, T_CM_ and transitional memory T (T_TM_; an intermediate cell type between T_CM_ and T_EM_ cells) cells are defined to be CD45RA^lo^CD27^hi^, T_EM_ cells are defined to be CD45RA^lo^CD27^lo^, and T_EMRA_ cells are defined to be CD45RA^hi^CD27^lo^.^42^

### Proliferation assay in PBMCs

A matched sample of 1 × 10^6^ PBMCs was stained with cell trace violet (CTV) dye (Life Technologies), as per the manufacturer's instructions, then stimulated with either αCD3 mAb or αCD3/CD28 mAbs with rhIL‐2, as described for the generation of CAR T cells. CTV‐stained, stimulated PBMCs were cultured at 37°C and 5% CO_2_ for 4 days. Cells were harvested, stained with LIVE/DEAD™ Near‐IR Dead Cell Stain (Life Technologies) for 10 min at room temperature, then stained with fluorescently conjugated anti‐human mAb against the following surface markers for 30 min at 4°C: TCR (clone IP26; AF700; eBiosciences), CD8 (SKI; SB600; eBiosciences), CD4 (SK3; APC; Tonbo Biosciences), CD45RA (5H9; FITC; BD Pharmingen), CD27 (L128; BV711; BD Pharmingen). The gating strategy is detailed in Supplementary figure 6.

### Intracellular cytokine staining in CAR T cells

To measure CAR‐specific cytokine production, CAR T cells were cocultured with iK562 nsHER2^−^ or iK562 nsHER2^+^ cells with rhIL‐2 (200 IU mL^−1^, Peprotech), GolgiPlug (1:1000; BD Biosciences) and αCD107a (H4A3; V450; BD Biosciences) for 16 h at 37°C and 5% CO_2_. Cells were stained with LIVE/DEAD™ Near‐IR Dead Cell Stain (Life Technologies) for 10 min at room temperature, then stained with fluorescently conjugated anti‐human mAb against the following surface markers for 30 min at 4°C: TCR (clone IP26; AF700; eBiosciences), CD8 (SKI; SB600; eBiosciences), CD4 (SK3; APC; Tonbo Biosciences). Cells were fixed and permeabilised using the Cytofix/Cytoperm kit (BD Pharmingen), as per the manufacturer's instructions, and then stained intracellularly for IFNγ (B27; FITC; BD Biosciences), IL‐2 (MQ1‐17H12; PE; BD Pharmingen), and TNF (Mab11; APC; BioLegend). Fluorescence minus one (FMO) controls were used to define cytokine gating and values for cytokine production are background subtracted. The gating strategy is detailed in Supplementary figure 7.

### Cytotoxicity assay

MDA‐468 cells expressing luciferase with (HER2^+^) or without HER2 (HER2^−^) were cultured in cRPMI for a minimum of 7 days prior to use in the cytotoxicity assay, then added to wells of a white‐walled 96‐well Culture Plate (PerkinElmer, Waltham, USA). CAR T cell samples were thawed overnight, CAR expression was assessed, CAR T cell numbers were equalised across samples and then added to the MDA‐468 cells at either 20:1, 10:1 or 1:1 effector to target (E:T) ratios. Co‐cultures were incubated for 16 h at 37°C and 5% CO_2_. The plate was washed twice with warm cRPMI followed by addition of 100 μL/well D‐luciferin (150 μg mL^−1^; Merck, Darmstadt, Germany and immediate measurement of luminescence on the EnSpire® Multimode Plate Reader (PerkinElmer)). Loss of luminescence was used to detect the lysis of target cells and standardised to complete lysis using a positive control of tumor cells with the addition of 5% sodium dodecyl sulphate. Percent HER2‐specific lysis was determined by (i) calculating the proportion of luciferase reduction with MDA‐468 HER2^+^ cells, then (ii) calculating the proportion of luciferase reduction with MDA‐468 HER2^−^ cells, then (iii) subtracting the value for MDA‐468 HER2^−^ cells from MDA‐468 HER2^+^ cells.

### Data analysis, statistical analysis and novel metrics

All flow data were acquired on a BD LSRFortessa flow cytometer (Becton Dickinson) and analysed in FlowJo (version 10.8; FlowJo LCC, Ashland, USA). Statistical analysis was performed in GraphPad Prism or SPICE using standard non‐parametric tests, either the Mann–Whitney *U*‐test for non‐paired data (comparison of age groups) or Wilcoxon signed rank sum test for paired data (comparison of activation protocols) with Bonferroni correction for multiple comparisons, or Simple Linear Regression (Pearson correlation coefficients for correlative studies), as indicated in the figure captions.

The differentiation score was calculated as (the frequency of T cells that are T_N_ × 1) + (the frequency of T cells that are T_CM_/T_TM_ × 3) + (the frequency of T cells that are T_EM_ × 5) + (the frequency of T cells that are T_EMRA_ × 7).

## Author contributions

**Palak H Mehta:** Conceptualization; data curation; formal analysis; investigation; methodology; project administration; validation; visualization; writing – original draft; writing – review and editing. **Gemma S Trollope:** Investigation; methodology. **Patrick Leung:** Investigation; methodology. **Shivali Savita Chinni:** Investigation; methodology. **Anna Iasinskaia:** Investigation; methodology. **Aaron J Harrison:** Investigation; methodology; resources. **Hannah Hughes‐Parry:** Investigation; methodology; resources. **Misty R Jenkins:** Investigation; methodology; resources; writing – review and editing. **Michael H Kershaw:** Conceptualization; funding acquisition; investigation; methodology; resources. **Anthony Jaworowski:** Conceptualization; methodology; supervision; writing – review and editing. **Clare Y Slaney:** Conceptualization; funding acquisition; investigation; methodology; resources; writing – review and editing. **Rachel M Koldej:** Conceptualization; data curation; investigation; methodology; project administration; resources; supervision; writing – review and editing. **David S Ritchie:** Conceptualization; funding acquisition; investigation; methodology; project administration; resources; supervision; writing – review and editing. **Kylie M Quinn:** Conceptualization; data curation; formal analysis; funding acquisition; investigation; methodology; project administration; supervision; validation; visualization; writing – original draft; writing – review and editing.

## Conflict of interest

The authors declare no conflict of interest.

## Supporting information


Supplementary figure 1
Supplementary figure 2
Supplementary figure 3
Supplementary figure 4
Supplementary figure 5
Supplementary figure 6
Supplementary figure 7

## Data Availability

The data that support the findings of this study are available from the corresponding author upon reasonable request.
