# Supplementary material for: Choice of activation protocol impacts the yield and quality of CAR T cell product, particularly with older individuals
Source: Clin Transl Immunology. 2024 Nov 29;13(12):e70016. doi: 10.1002/cti2.70016 (PMC11605362; doi:10.1002/cti2.70016)
Supplement: Supplementary file 1 — Supplementary figure 1 Supplementary figure 2 Supplementary figure 3 Supplementary figure 4 Supplementary figure 5 Supplementary figure 6 Supplementary figure 7 [file CTI2-13-e70016-s001.docx]

**SUPPORTING INFORMATION**

**SUPPLEMENTARY FIGURES**

**Supplementary figure 1:** Frequency of CAR expressing T cells. Mean number of divisions of **(a)** CD8 or **(b)** CD4 T cells from all donors, or young *vs* older donors at day 4 post activation. Frequency of **(c)** CD8 or **(d)** CD4 T cells that are CAR+ from all donors, or young vs older donors at day 10 post activation. Bars indicate mean ± SEM, and symbols on bar graphs indicate donors. * indicates *P* ≤ 0.0166 using the Mann-Whitney *U*-test (between donor groups) or pairwise Wilcoxon test (between activation protocols) with Bonferroni correction for multiple comparisons. If *P* ≤ 0.05, we have indicated this on the graphs.

**
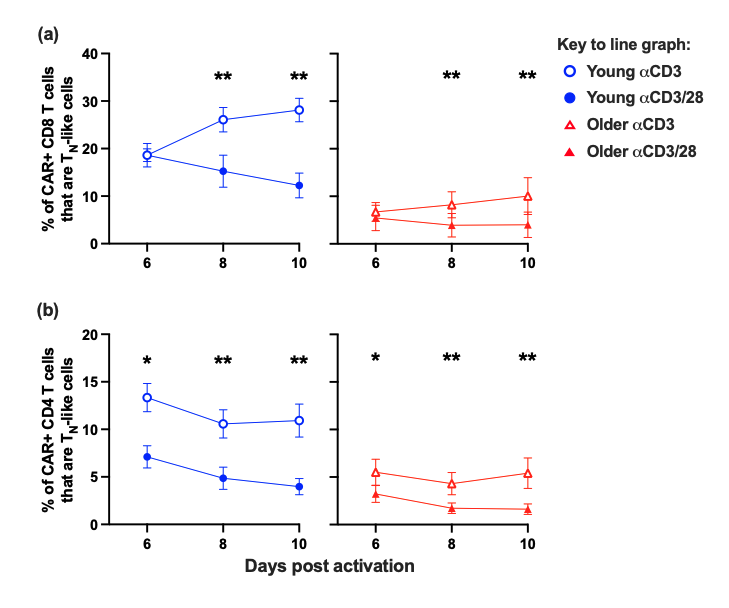
**

**Supplementary figure 2:** Expansion of T_N_-like CAR T cells. Frequency of **(a)** CD8 and **(b)** CD4 CAR T cells that are T_N_-like at day 6, 8 and 10 after activation. Bars indicate mean ± SEM, symbols indicate donors, * indicates *P* < 0.05, ** indicates *P* < 0.01, using the Mann-Whitney *U*-test (between donor groups).

**Supplementary figure 3:** Impact of activation protocol and age on the cytokine production of CD4 CAR T cells. **(a)** The frequency of CD4 CAR T cells that express IFNγ, TNF or IL-2 in response to HER2. **(b)** The frequency of CD4 CAR T cells that express any combination of IFNγ, TNF and/or IL-2 in response to HER2. Bars indicate mean ± SEM, symbols indicate donors. No significant differences were observed using the Mann-Whitney *U*-test (between donor groups) or pairwise Wilcoxon test (between activation protocols) with Bonferroni correction for multiple comparisons.

**Supplementary figure 4:** CD28 expression on T cell subsets from PBMC samples isolated from young and older healthy donors. Frequency of **(a)** CD8 or **(b)** CD4 T cells that express CD28 in each indicated subset. CD28 MFI on CD28^+^ **(c)** CD8 and **(d)** CD4 T cell subsets. Bars indicate mean ± SEM, symbols indicate donors, * indicates *P* < 0.05, using the Mann-Whitney *U*-test (between donor groups)**.**

**Supplementary figure 5:** Flow cytometry gating strategy to identify T cell phenotypes and CD28 expression in PBMCs (shown) and CAR T cells.

**Supplementary figure 6:** Flow cytometry gating strategy for CTV analysis of T cell proliferation at day 4 after activation.

**Supplementary figure 7:** Flow cytometry gating strategy to identify cytokine production by CAR T cells after co-culture with iK562HER2^+/-^ cells for 16 hours.
